# Correction: Dynamic expression of cathepsin L in the black soldier fly (*Hermetia illucens*) gut during *Escherichia coli* challenge

**DOI:** 10.1371/journal.pone.0334338

**Published:** 2025-10-10

**Authors:** Yun-Ru Chiang, Han-Tso Lin, Chao-Wei Chang, Shih-Ming Lin, John Han-You Lin

The images for Figs 4 to 7 are incorrectly switched. The image that appears as Fig 4 should be Fig 5, the image that appears as Fig 5 should be Fig 6, the image that appears as Fig 6 should be Fig 7 and the image that appears as Fig 7 should be Fig 4. The figure captions appear in the correct order. The authors have provided a corrected version of figures here.

**Fig 4:**
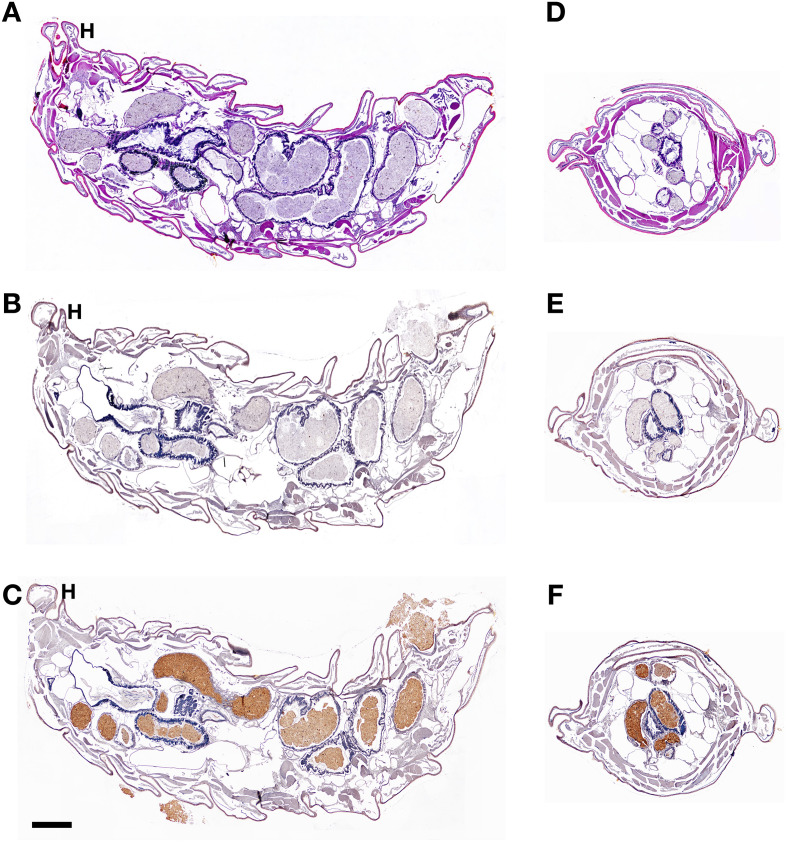
Morphological organization and immunohistochemical localization of CTSL in BSFL. The histology of BSFL is presented in longitudinal sections **(A, B, C)** and cross sections **(D, E, F)**. H&E staining revealed the main structure of the fifth instar of BSFL **(A, D)**. BSFL were stained with naïve mouse serum as a negative control **(B, E)**. BSFL were stained by chromogenic IHC methods following injection of mice anti-BSFL CTSL serum **(C, F)**. Positive signals were revealed in brown, while hematoxylin was used as a counterstain. H, head. (Bar = 500 μm).

**Fig 5:**
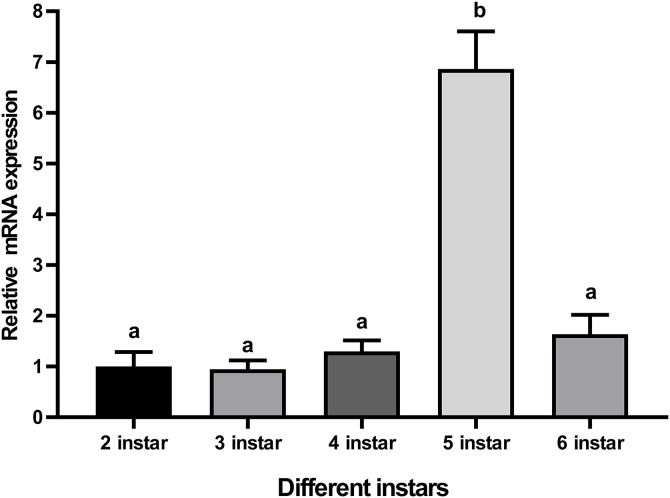
Relative expression levels of CTSL mRNA in the different larval stages. Relative mRNA expression (2 ^-ΔΔct^) levels of CTSL were detected using SRBR green assay and normalized to β-actin. The fold-change of CTSL expression was calculated for the 3^rd^, 4^th^, 5^th^, and 6^th^ instars and compared with the 2^nd^ instar. Bars represent mean ± S.E. (n = 3). Letters indicate significant differences (*p* < 0.05).

**Fig 6:**
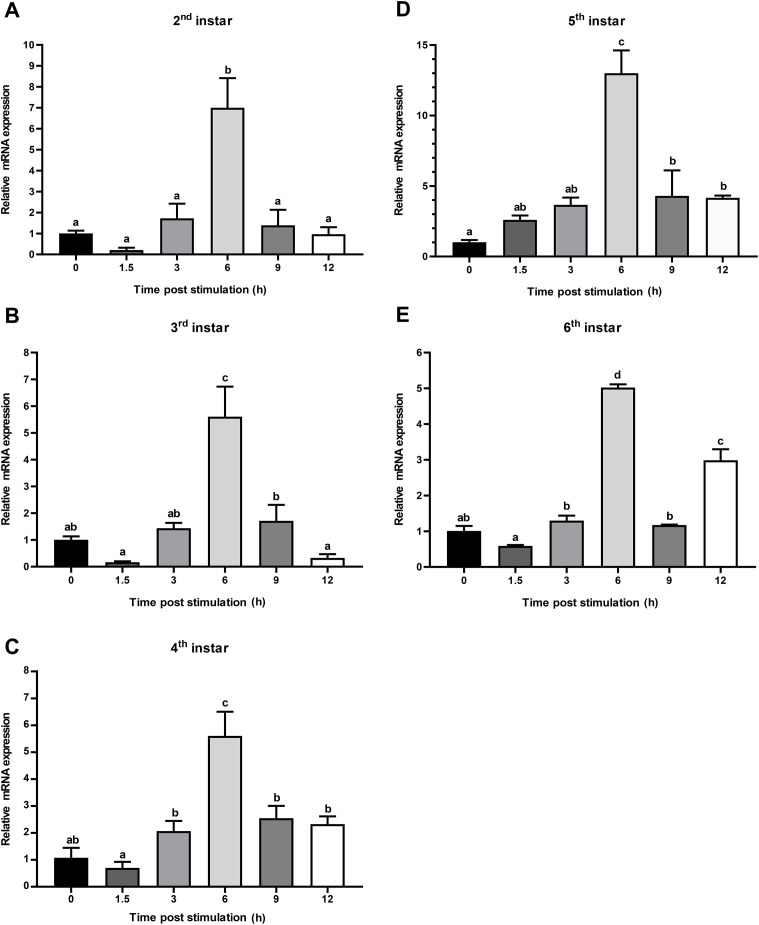
Dynamic expression of CTSL mRNA in response to *E*. *coli* challenge. Relative mRNA expression (2 ^-ΔΔct^) levels of CTSL were detected using SRBR green assay and normalized to β-actin in the 2^nd^ **(A)**, 3^rd^ **(B)**, 4^th^ **(C)**, 5^th^ **(D)**, and 6^th^ (E) instars. The fold-change of CTSL expression was calculated at 1.5, 3, 6, 9, and 12 h post-stimulation with *E*. *coli* and compared with 0 h (negative control was non-treated BSFL). Bars represent mean ± S.E. (n = 3). Statistical significance is indicated with lowercase letters (*p* < 0.05).

**Fig 7:**
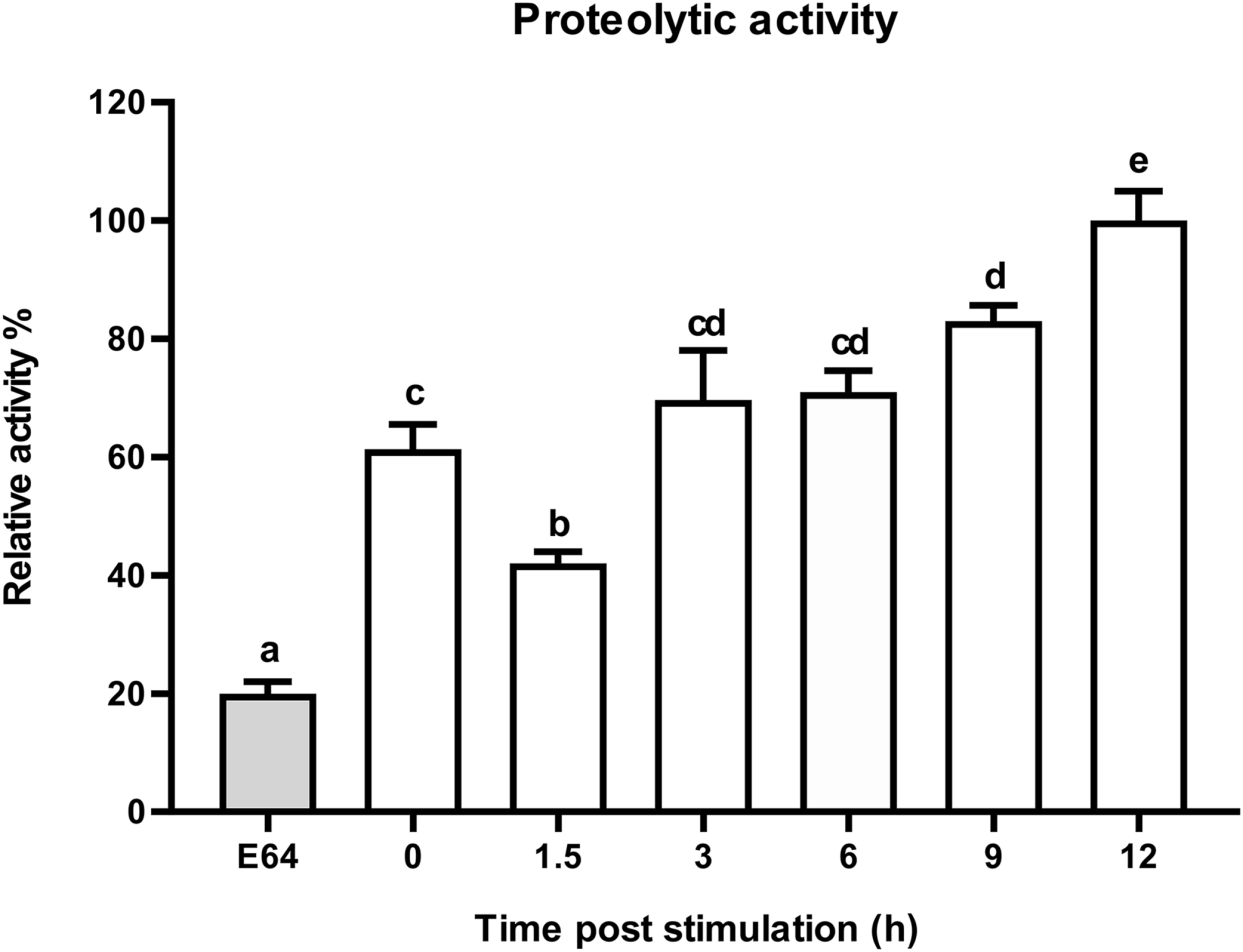
Dynamic proteolytic activity of CTSL in response to *E*. *coli* challenge. Percentage of CTSL activity in the fifth instar under *E*. *coli* challenge compared to negative control (cysteine proteinase inhibitor E-64). Error bars represent mean ± S.E. (n = 5). Statistical significance is indicated with different lowercase letters (*p* < 0.05).
