# Supplementary figures and images for: Dynamic expression of cathepsin L in the black soldier fly (Hermetia illucens) gut during Escherichia coli challenge
Source: PLoS One. 2024 Mar 7;19(3):e0298338. doi: 10.1371/journal.pone.0298338 (PMC10919656; doi:10.1371/journal.pone.0298338)

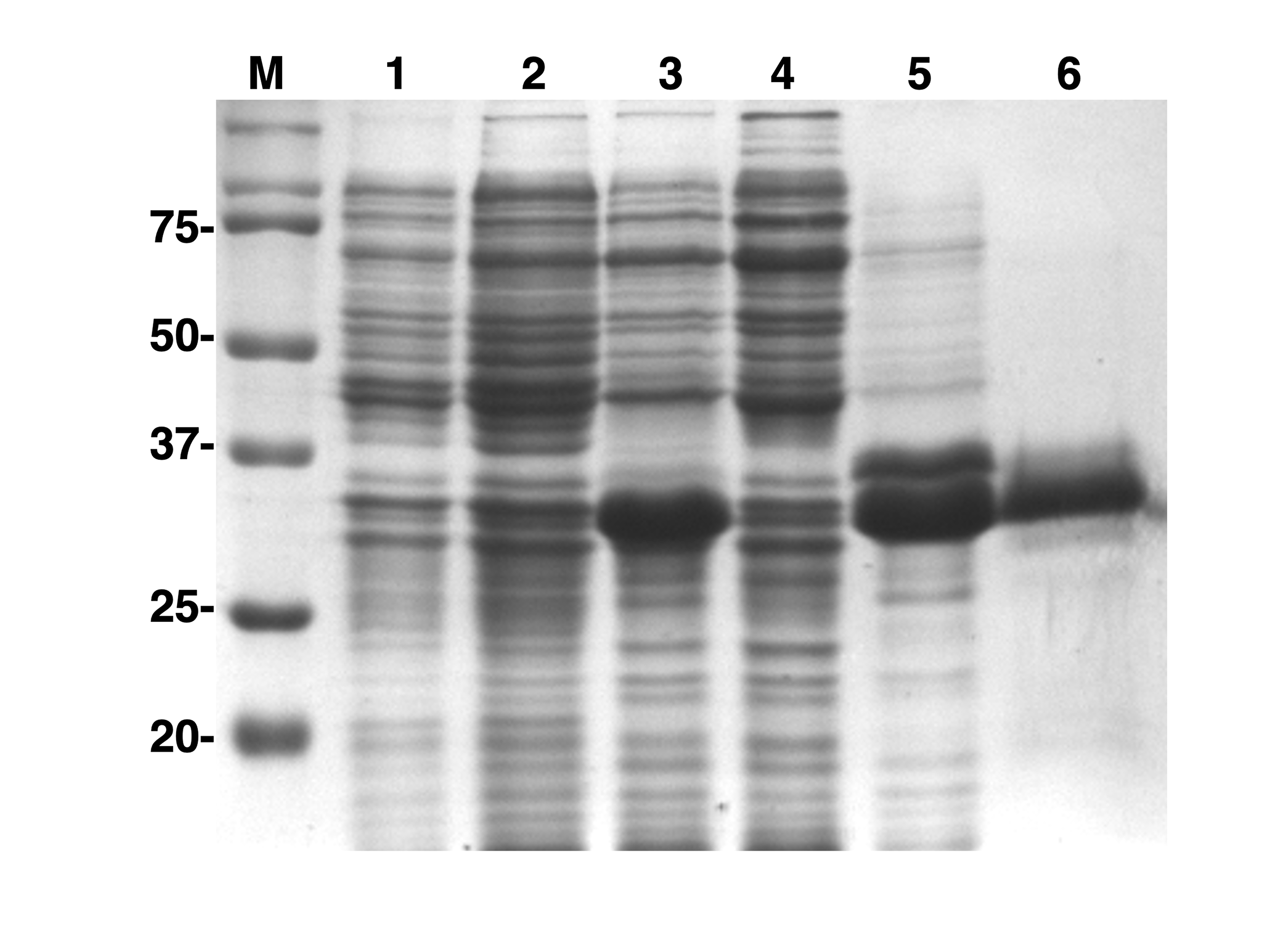

Supplement: S1 Fig — M: Protein molecular weight marker; lane 1, total protein extracted from E. coli BL21 (DE3); lane 2: E. coli containing pET-24a-CTSL without IPTG induction; lane 3: E. coli containing pET-24a-CTSL with IPTG induction; lane 4: soluble protein; lane 5: inclusion bodies; lane 6: purified recombinant CTSL protein. (TIF) [file pone.0298338.s001.tif]

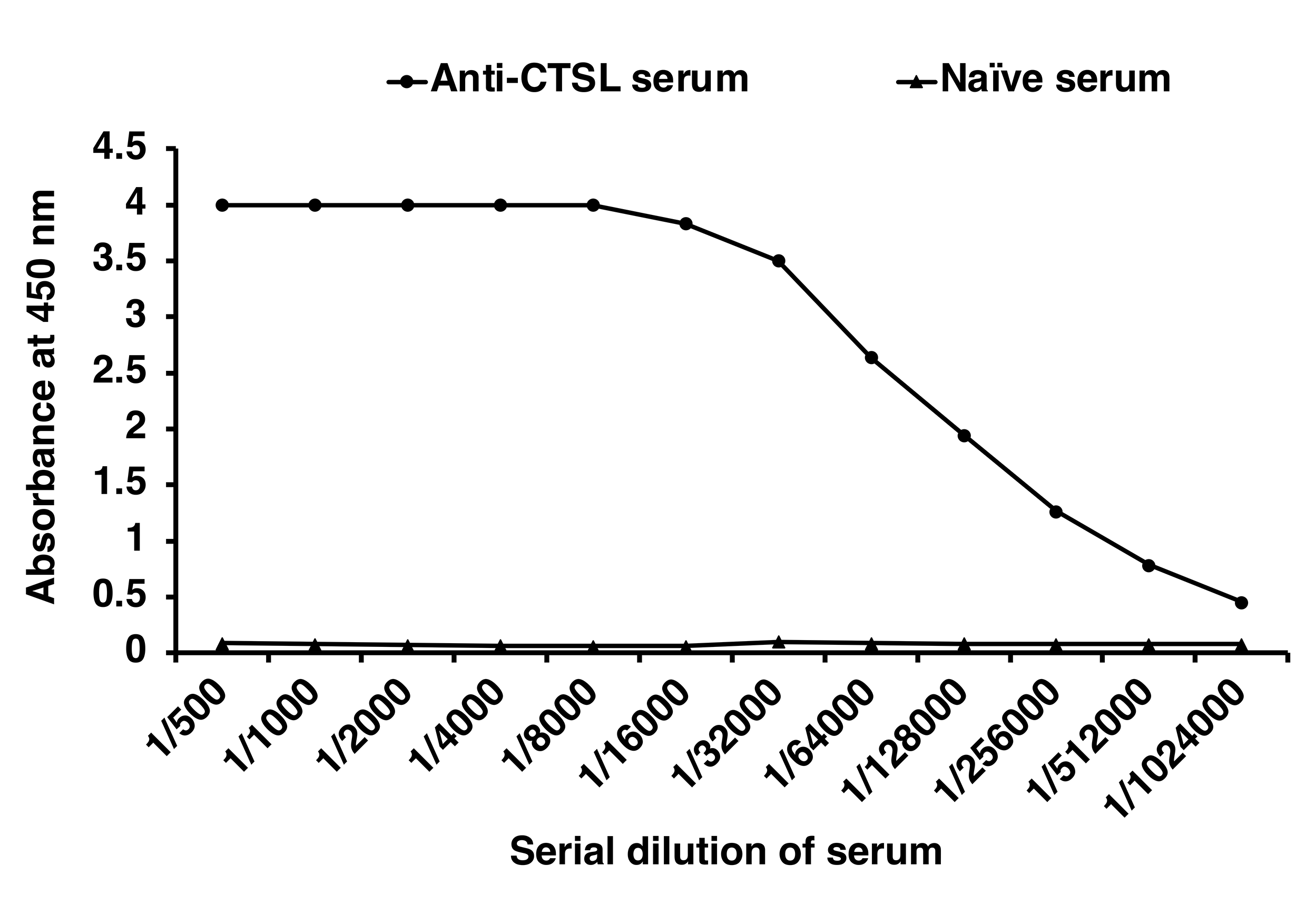

Supplement: S2 Fig — After the final immunization, antiserum was serially diluted, and the absorbance values were measured. (TIF) [file pone.0298338.s002.tif]
