# Supplementary material for: Dynamic expression of cathepsin L in the black soldier fly (Hermetia illucens) gut during Escherichia coli challenge
Source: PLoS One. 2024 Mar 7;19(3):e0298338. doi: 10.1371/journal.pone.0298338 (PMC10919656; doi:10.1371/journal.pone.0298338)
Supplement: Attachment — Submitted filename: PLOS one Reviewer_v4.docx [file pone.0298338.s003.docx]

**Reviewer #1:**

Line 41, please add references (for example <https://doi.org/10.1111/1744-7917.13155>, Bulletin of Insectology 75 (1): 75-82, 2022)

Response: Thank you for the suggestion. The appropriated references have been cited in line 41.

Line 63, please add more recent references (for example <https://doi.org/10.3390/insects14050464>)

Response: Thank you for the suggestion. The appropriated references have been cited from line 63-65.

Line 73, please insert a reference

Response: Thank you for the suggestion. The appropriated references have been cited in line 74.

**MATERIAL AND METHODS**

Line 120-125 Description of BSF is useless

Response: The description has been modified in line 122 to 123.

Line 186: it is necessary to add the concentrations used

Response:

Thank you for the suggestion. The concentrations of ethanol have been added in line 183.

Line 190: it is necessary to add the concentrations used

Response:

Thank you for the suggestion. The concentrations of ethanol have been added in line 187.

**Reviewer #2:**

Chiang et al describe expression of cathepsin L in black soldier fly larvae (BSFL) while challenged by infection of *E. coli*.
The overall conclusion that under infection levels of CTSL are elevated and that this provides support for the potential role of CTSL in immunological response of BSFL in the gut are supported by results. Therefore, the ms is to be published. There are however a few concerns, which should be addressed prior acceptance.
The RNA and immune analysis are consistent and refer to the specific protein, called CTSL. This however can not be claimed for the measurements of proteolytic activity.
The Z-Phe-Arg-AMC substrate used is not as specific for cathepsin L as the authors believe. It can be used on other cathepsin as well. Furthermore, this substrate was developed to be used on mammalian cathepsins. As their sequence analysis shows the BSF CTLS has 50% identity with human CTSL. The indicated difference already indicates that the substrate is not as specific. Therefore I suggest the authors to comment the following issues:

- How similar is BSF CTSL from other human cysteine cathepsins? Is the human CTSL indeed its closest human homologue?

Response:

Sequence similarity is crucial for protein function, but the protein function is ultimately determined by the catalyst center. Despite BSF CTSL sharing only 50.29% amino acid identity with human CTSL, the catalytic active sites of BSF CTSL, including Cys, His, and Asn (Responses figure 2), are the same as those in human CTSL. Moreover, the 3D structures are similar. When the protein 3D structure was analyzed using SWISS-MODEL, the GMQE was up to 0.97 with *Drosophila guanche* (Fruit fly) CTSL, and up to 0.80 with human CTSL (Responses figure 1), indicating a high coverage dependency between BSF CTSL, Fruit fly CTSL, and human CTSL. This similarity implies that BSF CTSL may exhibit similar proteolytic activity to fruit fly CTSL and human CTSL. Additionally, the Z-Phe-Arg-AMC served as the subtract to demonstrated enzymatic activity of CTSL from *Delia coarctata* [1] and *Sarcophaga peregrin*a [2]. The appropriated references have been cited in line 244. Based on these evidences, we suggest that BSF CTSL can also cleave the substrate by attacking the peptide bond between arginine and AMC.

Responses figure 1. BSF CTLS was analyzed by SWISS-MODEL. (A) The score of GMQE and QMEAND. (B) The amino acid sequence of BSF CTLS aligned with human CTSL.

Responses figure 2. The catalytic active sites of BSF CTLS were shown in the 3D model.

- Is CTSL the only cathepsin-like enzyme in the BSF genome? Other species have multiple cathepsin. How much is known about this?
Namely, the kinetic data could have recorded also activities or other cysteine cathepsins, which implies that also levels of other cysteine cathepsins are elevated during the challenge.

Response:

Unlike humans, who possess 11 cysteine cathepsins, only five cysteine cathepsins (cathepsin B, D, F, L, and O) have been reported in insects [3]. Previous studies did not provide detailed characteristics of cathepsins in BSFL. This study represents the first exploration of cathepsins in BSFL. As we know, Z-Phe-Arg-AMC serves as a substrate for papain, trypsin, and some cysteine cathepsins. However, it is challenging to identify the specific cysteine cathepsins involved in enzyme catalysis from the BSFL extract. In our study, the IHC data, using specific antibodies, demonstrated that the protein level of CTSL is abundant in the BSFL gut. Moreover, CTSL mRNA expression increased after 3 h post E. coli stimulation. This profile paralleled the observed proteolytic activity, which also showed a gradual increase after 3 h after *E. coli* stimulation. Although it was challenging to prove that Z-Phe-Arg-AMC is exclusively cleaved by CTSL, our observations suggest that CTSL abundant expression in the body. Therefore, CTSL might play a crucial role in the trial involving the cleavage of Z-Phe-Arg-AMC. However, further investigation is required to substantiate this perspective. (See Lines 362-369 in the revised manuscript)

- It is not obvious to me the relationship between the needle injury and immune response, as the observed CTSL was mostly elevated in guts tissue, which could have come in contact with *E. coli* also during the feeding process. Please elaborate this.

Response:

The mechanism through which needle injury triggers the immune system in BSFL remains unclear. According to previous studies, when larvae were punctured with a sterile needle and subsequently exposed to live bacteria, there was an increase in both antimicrobial peptides (AMPs) and defensin-like peptides [4, 5]. The combined effect of needle injury and bacterial exposure has been observed to activate innate immune response pathways in BSFL.

In our previous data (Responses figure 3), BSFL were treated in five different conditions: First, no treatment served as the negative control; second, wounds were inflicted using needles (wound only); third, no wounds were made, but the larvae were immersed in *E. coli* suspension; forth, wounds were inflicted, and larvae were soaked in LB; fifth, wounds were inflicted, and larvae were dipped into a suspension of *E. coli*. The results showed no significant difference in CTSL among the first four stimulative conditions. This finding suggested even when BSFL comes into contact with *E. coli* during the feeding process, the expression of CTSL is not influenced. However, when we mimicked the *E. coli* invading process by needle injury and immersed BSFL in *E. coli*, the expression of CTSL 6 hours post-invasion was 13 times higher than that of the control. This implies that *E. coli* could invade through a wound, leading to an increase in CTSL expression.

Based on previous studies and our observations, we suggest that this method has the potential to trigger suitable innate immune responses.

**Responses figure 3. CTSL mRNA expression in different treatments of BSFL.** The mRNA expression was normalized to levels of the β-actin housekeeping gene. Bars represent mean ±S.E. (n=3). Different letters indicate statistically significant differences (p < 0.05) in different treatments.

References

1.	Pyati PS, Bell HA, Fitches E, Price DR, Gatehouse AM, Gatehouse JA. Cathepsin L-like cysteine proteinase (DcCathL) from Delia coarctata (wheat bulb fly): basis of insecticidal activity. Insect biochemistry and molecular biology. 2009;39(8):535-46.

2.	Philip JM, Fitches E, Harrison RL, Bonning B, Gatehouse JA. Characterisation of functional and insecticidal properties of a recombinant cathepsin L-like proteinase from flesh fly (Sarcophaga peregrina), which plays a role in differentiation of imaginal discs. Insect biochemistry and molecular biology. 2007;37(6):589-600.

3.	Saikhedkar N, Summanwar A, Joshi R, Giri A. Cathepsins of lepidopteran insects: Aspects and prospects. Insect biochemistry and molecular biology. 2015;64:51-9.

4.	Nakagawa A, Sakamoto T, Kanost MR, Tabunoki H. Development of new methods to stimulate the production of antimicrobial peptides in the larvae of the black soldier fly Hermetia illucens. 2023.

5.	Zdybicka-Barabas A, Bulak P, Polakowski C, Bieganowski A, Waśko A, Cytryńska M. Immune response in the larvae of the black soldier fly Hermetia illucens. Invertebrate Survival Journal. 2017;14(1):9-17.
